# Investigating the Symptom Presentation of Depression in Children with ADHD

**DOI:** 10.1192/bjo.2025.10246

**Published:** 2025-06-20

**Authors:** Gareth Williams, Victoria Powell, Olga Eyre, Anita Thapar, Lucy Riglin

**Affiliations:** Wolfson Centre for Young People’s Mental Health and Centre for Neuropsychiatric Genetics and Genomics, Division of Psychological Medicine and Clinical Neurosciences, Cardiff University, Cardiff, United Kingdom

## Abstract

**Aims:** ADHD is commonly comorbid with depression and this comorbidity is associated with increased symptom severity and worse outcomes than either condition alone. Depression is highly heterogeneous and may present differently in populations with ADHD. This study aimed to explore different symptom presentations of depression and associated clinical correlates in a clinical ADHD sample.

**Methods:** We analysed data from the Study of ADHD Genes and Environment (SAGE). Parents completed semi-structured questionnaires about their child’s psychopathology at baseline (mean age 10.9 years) and the Mood and Feelings Questionnaire to capture their child’s depression symptoms approximately 5 years later (mean age = 14.6 years, N=249). Depression symptom presentations were derived by latent profile analysis.

**Results:** Analyses found three presentations of depression symptoms: a ‘low symptoms’ (48.5% of the sample) class, a ‘high symptoms’ class (15.5%) with consistently high depression symptoms, particularly for suicidality and poor self-esteem items, and an ‘irritable/poor sleep’ class (36.1%) with intermediate levels of depression symptoms and high scores for irritability and poor sleep. All three classes had elevated irritability and symptoms that overlap with ADHD. Behavioural problems were associated with an increased likelihood of being in the high symptoms compared with low symptoms class, and higher autism symptoms were associated with being in the intermediate ‘irritable/poor sleep’ compared with low class.

**Conclusion:** Our findings suggest that while young people with ADHD often have elevated depression symptoms, there is notable heterogeneity. Young people with ADHD and behavioural disorders may be particularly at risk of more severe depression symptom presentations characterised by high suicidal cognitions, whilst those with ADHD and autistic traits may present with more irritability and poor sleep.

